# Loss to follow-up of HIV-exposed infants for confirmatory HIV test under Early Infant Diagnosis program in India: analysis of national-level data from reference laboratories

**DOI:** 10.1186/s12887-022-03656-w

**Published:** 2022-10-18

**Authors:** Nilesh Gawde, Suchit Kamble, Noopur Goel, Kalyani Nikhare, Shilpa Bembalkar, Mohan Thorwat, Dhanashree Jagtap, Swarali Kurle, Neeru Yadav, Vinita Verma, Neha Kapoor, Chinmoyee Das

**Affiliations:** 1 Tata Institute of Social Sciences, Mumbai, India; 2 ICMR - National AIDS Research Institute, Pune, India; 3 ICMR – National Institute for Research in Reproductive Health, Mumbai, India; 4 National AIDS Control Organisation, New Delhi, India

**Keywords:** Early infant diagnosis, HIV-exposed infants, Loss to follow-up from EID testing cascade

## Abstract

**Background:**

Early Infant Diagnosis was launched in India in 2010 and its effect on the diagnosis of HIV-exposed infants needs to be assessed. The present study was done to find out the median age at DBS sample collection for early infant diagnosis and its trend over years, the median age at diagnosis of HIV among the HIV-exposed infants with DNA PCR tests, and the proportion of infants who completed testing cascades after detection of HIV-1 in a sample.

**Methods:**

DNA PCR data (from 2013 to 2017) maintained at all regional reference laboratories in India was collated with each infant identified by a unique code. Cohort analysis of the infant data was used to find the median age at sample collection and diagnosis. The outcomes of testing in each cascade and the overall outcomes of testing for infants were prepared.

**Results:**

The median age at sample collection for the four years combined at all India level was 60 days (48–110 days). The median age at diagnosis of HIV was 285 days (174–418 days). HIV-1 was detected in samples of 1897 (6.3%) infants out of 30,216 infants who had a DNA PCR test, out of whom 1070 (56.4%) completed the testing cascade and the rest were lost to follow-up.

**Conclusion:**

The data highlights delay in diagnosis; both due to delay in sample collection and turn-around-times. Loss to follow-up of HIV-exposed infants with virus detection is a significant concern to the Early Infant Diagnosis and tracking systems need to be strengthened.

**Supplementary Information:**

The online version contains supplementary material available at 10.1186/s12887-022-03656-w.

## Background

Perinatally acquired Human Immunodeficiency Virus (HIV) infection has posed considerable challenges to concerning its prevention, diagnosis, and treatment in program settings globally. Mortality can be reduced by three-fourths with early initiation of Antiretroviral Treatment (ART) among such infants [1]. More than a decade has passed since the efficacy studies but access to ART remains poor and delay in confirmed HIV diagnosis is the crucial underlying reason. Most nations adopted the World Health Organisation (WHO) recommendation of early infant diagnosis (EID) of HIV but the reach of EID services remains poor. Several studies from sub-Saharan Africa have identified the gaps within the health systems such as delay in transport, the long turnaround time for diagnosis, and loss to follow-up [2]. Over the past decade, the scale of services has improved but the health systems challenges have persisted limiting access and impact [3].

India continues to be a low prevalence epidemic country and the National AIDS Control Programme (NACP) is able to detect about 15,000 HIV-positive pregnant women annually out of the estimated 22,000 [4]. Early Infant Diagnosis (EID) services were launched a decade ago through seven regional reference laboratories (RRL) which cover the vast geography of the nation. The services of sample collection are offered at selected stand-alone integrated counseling and testing centers (SA-ICTCs) at the grass root level. Dried blood spot (DBS) samples are transported to one of the seven RRLs where Deoxyribonucleic Acid Polymerase Chain Reaction (DNA PCR) tests are conducted. Detection of HIV-1 in two separate samples forms the basis of diagnosis under 18 months of age. The EID service consists of the complex algorithm in terms of collection of samples at scheduled periods of 6 weeks, 6 months, and 12 months, collection of DBS for detecting virus, transportation of DBS samples to RRL, communication of test results to ICTC and then to the caregiver, collection of the second DBS sample for confirmation of diagnosis (for cases with virus detection in the first sample) and subsequent follow-up for further testing till 18 months of age. Routine program reporting includes a number of infants who accessed EID services but does not include analysis of cascade completion rates and age at HIV diagnosis. Understanding the testing cascade completion rates and age at diagnosis is critical for the program. Previous studies have examined data from a single facility or a state [5, 6], and very few have examined cascade completions. This is the first paper presenting nationwide data of the age at HIV diagnosis and the EID cascade completion rates among HIV-exposed infants from all reference laboratories in India.

## Materials and methods

We conducted a retrospective analysis of data collected under the NACP about the EID services. The DNA PCR testing for detection of HIV-1 among samples of HIV-exposed infants is made available at seven RRLs that have digitized and stored data for each test. The DNA PCR tests at these RRLs were conducted using the Amplicor ® HIV-1 DNA Test, version 1.5 by Roche, USA on the DBS specimens. Each HIV-exposed infant is given a unique ID when the sample is collected first time for the DNA PCR test. All the subsequent DNA PCR samples from that infant are tagged with the same unique ID. Data from all seven laboratories from the year 2013 to 2017 was pooled. This dataset included some samples of infants born before 2013 whose samples were collected in the evaluation period and such data was removed before analysis. Thus, the final dataset included DNA PCR test data of HIV-exposed infants born from 2013 to 2016.

**Fig. 1:**
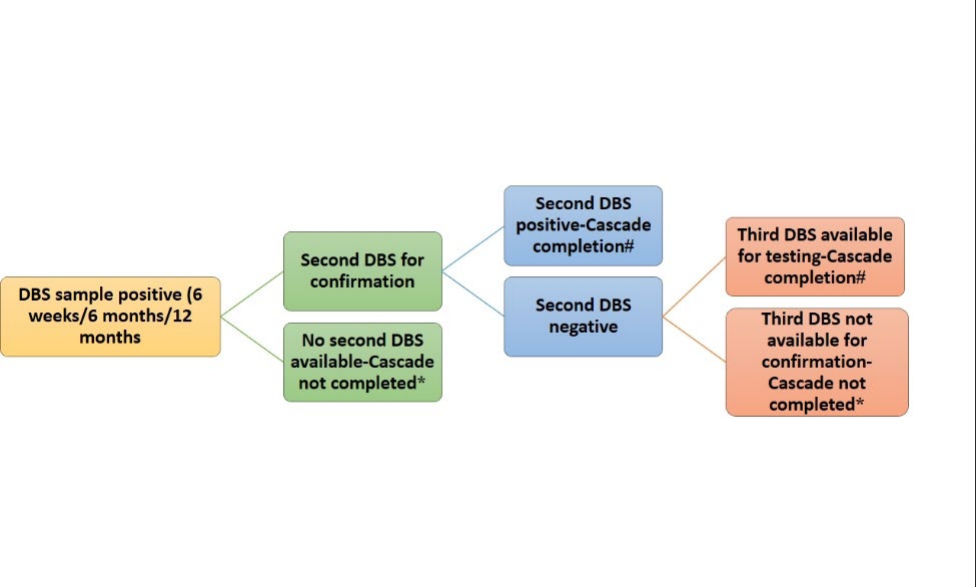
Conceptual figure for EID testing cascade completion. * Cascade not completed, # Cascade completion, DBS: Dried Blood Sample

Under NACP, periodic samples are to be collected at age of 6 weeks, 6 months and 12 months at the stand-alone ICTC (SA-ICTC). If the child is less than 6 months old, then a dried blood spot (DBS) sample is collected for a DNA PCR test. For infants aged 6 to 18 months, initially, antibody tests are performed at the SA-ICTC, and samples for DNA PCR tests are collected only among those who had reactive antibody tests. If HIV-1 is detected on the initial DBS sample at any period, the second DBS sample is collected for a confirmatory test. HIV diagnosis is confirmed as positive if the second sample is also detected as positive. A third sample is needed if there is a discordant result in the first and second sample. Operationally a testing cascade was considered to be completed only if the second (repeat) DBS sample is found to be positive or if the second DBS sample is found to be negative and the third DBS sample is available for testing (Fig. 1). If the second sample for confirmatory test is not available or the third sample is not available if discordance result for first two samples, then the testing cascade was considered as incomplete. Though the periodic sample collection has been specified at a particular age by the program, a child may or may not report on that age. Therefore, children of age 6 weeks and above and reporting before 18 months of age for the first time were considered under the first testing cascade. If HIV status is negative under the first testing cascade and the child reported for further follow-up testing before 18 months of age, it was considered under the second testing cascade. If HIV status is negative under both the first and second testing cascade and the child reported for further follow-up testing before 18 months of age, it was considered under the third testing cascade. Once the HIV diagnosis has been confirmed as positive on two subsequent DBS samples, the baby is linked with the treatment centre and only the antibody test is done at the age of 18 months at the ICTC level. These conformed HIV-positive babies were excluded from further testing cascades. The RRL dataset included DNA PCR test data but not antibody tests conducted at SA-ICTCs.

Age at the collection of first DBS sample collection under testing cascade 1 was computed from the date of birth and the date of first sample collection for the DNA PCR test. Time trends were created for birth cohorts from 2013 to 2016. The mean age at diagnosis was computed based on the date on which the second DNA PCR test detected HIV-1 in the infant’s sample. In the initial days of the HIV/AIDS epidemic in India, six states (later on seven states due to the division of Andhra Pradesh state) had a higher prevalence of HIV compared to other states and these were termed as ‘high prevalence’ states by NACP [7]. Five of the seven laboratories were located within these high prevalence states. Given these differences, we hypothesized that the high prevalence states would have better access to EID services. Since the testing would occur generally three times during the 18 months, we analysed the data separately for each cascade. Cascade completion rates were calculated based on the number of infants who completed each of the cascades.

## Results

**Fig. 2:**
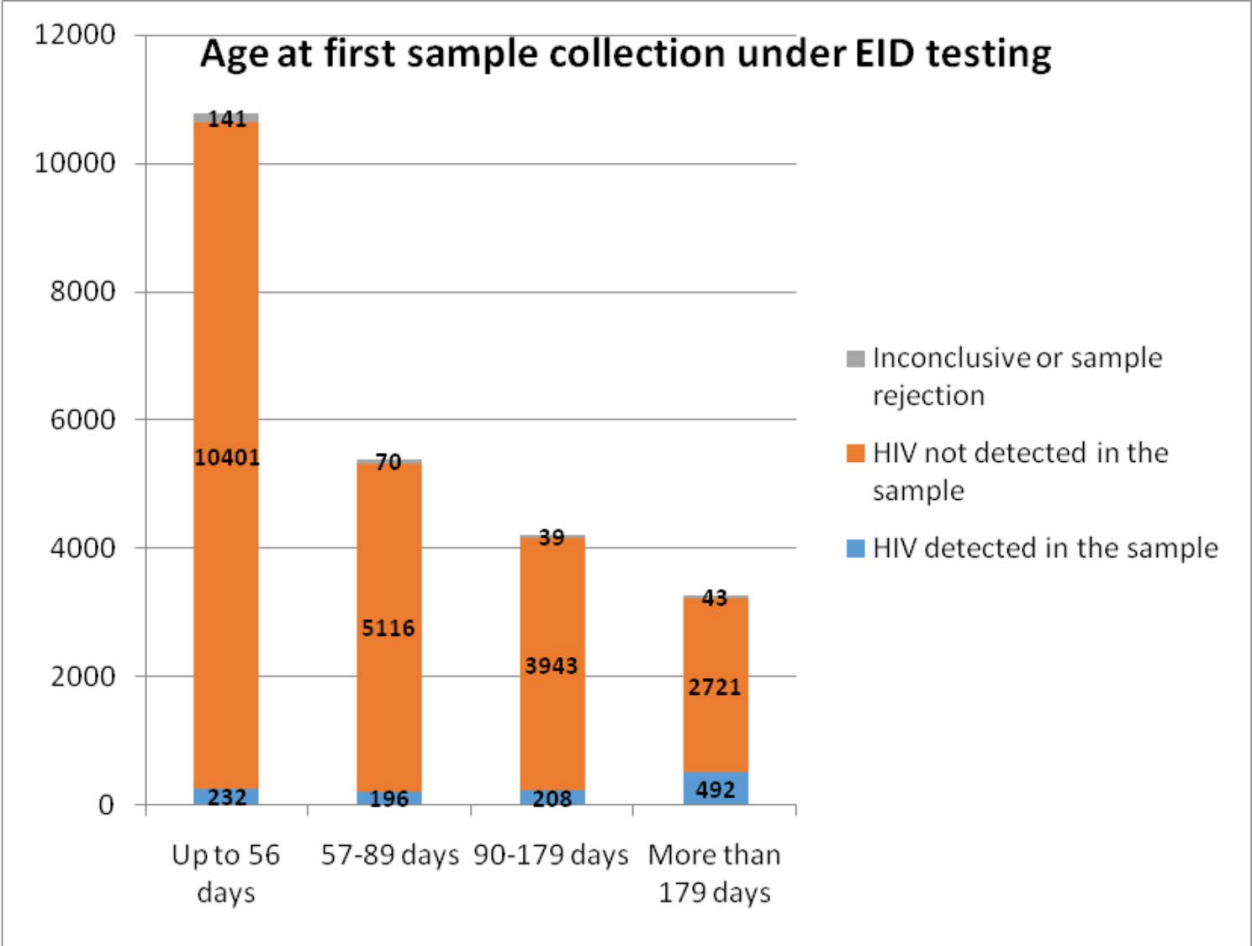
Age at the time of collection of first DNA PCR sample for children born from 2013 to 2016 (N = 23,602)

A total of 30,216 HIV-exposed infants’ samples were received by the seven regional reference laboratories during the study period. Out of these, birth dates were available only for 23,602 for the birth cohorts of 2013 to 2016. Under the 1st testing cascade, only 10,774 (45%) of the HIV-exposed infants were brought to the SA-ICTCs for sample collection within 8 weeks (56 days) of birth and another 5382 (23%) between 8 weeks to 3 months (57–89 days) (Fig. 2). Initial samples for 1st testing cascade were collected beyond the age of six months for 3256 (14%) infants. Table 1 shows that HIV was detected in 1128 (4.8%) infants’ initial samples under 1st testing cascade.

**Table 1:** Results of first DNA PCR tests under testing cascade 1 among HIV-exposed infants by age at the time of collection (N = 23,602)

Age at time of collection of first sample for DNA PCR test	Total Number of HIV-exposed infants	HIV detected in the sample	HIV not detected in the sample	Inconclusive or sample rejection
	N	No (%)	No (%)	No (%)
Up to 56 days	10,774	232 (2.2)	10,401 (96.5)	141 (1.3)
57–89 days	5382	196 (3.6)	5116 (95.1)	70 (1.3)
90–179 days	4190	208 (5.0)	3943 (94.1)	39 (0.9)
More than 179 days	3256	492 (15.1)	2721 (83.6)	43 (1.3)
Total	23,602	1128 (4.8)	22,181 (94.0)	293 (1.2)

**Fig. 3:**
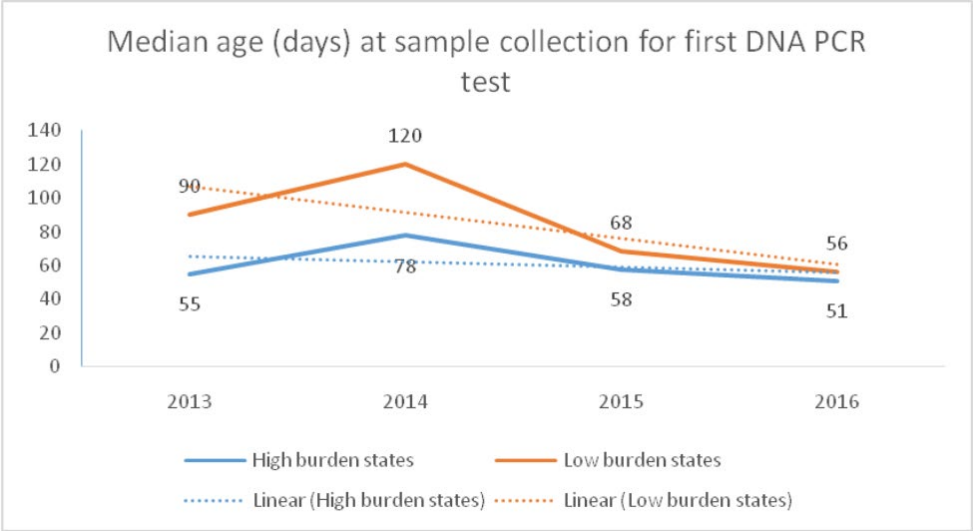
Median age (days) at the time of collection of first DNA PCR sample: Trend in high and low prevalence states, 2013-16 (N = 23,602). *High prevalence states include Maharashtra, Andhra Pradesh, Telangana, Karnataka, Tamil Nadu, Manipur and Nagaland; rest of the states and union territories are low-prevalence

Out of these 1128 infants, more than two third (68.5%) were from the seven Indian states. The median age at initial DBS sample collection under 1st testing cascade was declined over the period of four years (2013–2016) for both the high prevalence and low prevalence states (Fig. 3). High prevalence states consistently performed better compared to the low-prevalence states but the gap was closing by 2016. The median age at initial sample collection under the 1st testing cascade for the four years combined at all India level was 60 days (48–110 days).

The analysis for cascade completion was conducted for all 30,216 infants.

### Completion of 1st HIV testing cascade

HIV-1 was detected by DNA PCR test among 1576 (5.2%) infants’ initial samples entering the first cascade but out of which only 906 (57.5%) completed the first cascade with next sample/s (Table 2). Of the 30,216 infant samples, 28,539 (94.4%) had no detection of HIV-1; 18,315 (64.2%) did not have any further DNA PCR test whereas the rest 10,224 (35.8%) entered the second cascade along with 9 infants whose first DNA PCR test detected the virus but the next two confirmatory tests had failed to detect. Thus, 10,233(10,224 plus 9) entered the second DNA PCR cascade.

**Table 2:** Early Infant Diagnosis of HIV: completion of testing cascades, India

DNA PCR cascade	Total number of infants (a)	Out of a, HIV not detected (b)	Out of a, Inconclusive report (c)	Out of a, HIV detected in one sample (d)	Out of d, infants who completed the cascade (e)	Out of e, Number of infants diagnosed with HIV infection (f)
First cascade	30,216	28,539 (94.4)	101 (0.3)	1576 (5.2)	906 (57.5)	828 (91.4)
Second cascade	10,233	9916 (96.9)	69 (0.7)	248 (2.4)	130 (52.4)	105 (80.8)
Third cascade	2330	2233 (95.8)	24 (1.0)	73 (3.1)	34 (46.6)	27 (79.4)

### Completion of 2nd HIV testing cascade

Out of the 10,233 infants entering the second cascade, 248 (2.4%) had HIV-1 detected in their initial samples but only 130 (52.4%) completed the second cascade (confirmatory DBS sample was collected and tested); 9916 (96.9%) had no HIV-detected and 69 (0.7%) had inconclusive results. Out of the 9916, 2330 (23.5%) entered the third cascade and the rest had no more DNA PCR tests.

### Completion of 3rd HIV testing cascade

In the third cascade, out of 2330 infants, 73 (3.1%) had HIV-1 detected in their initial samples but only 34 (46.6%) reported further for confirmatory test and completed the 3rd cascade.

Overall, 1897 (6.3% of 30,216) infants had HIV-1 detected in the initial DNA PCR sample; 1576, 248 and 73 in the first, second and third cascade respectively. Of these 1897 infants, 1070 (56.4%) completed the respective cascade and the rest 827 (43.6%) did not complete the cascades.

Of these 1070 infants who completed the cascades for confirming the diagnosis, 960 (89.7%) were diagnosed with HIV infection; 949 with the second sample whereas only 11 (1.14%) needed a third sample (because the second sample did not detect HIV-1). The rates of confirmation of HIV diagnosis after initial detection were 91.4% (828/906) in first, 80.8% (105/130) in second and 79.4% (27/34) in the third cascade. Overall HIV-1 was not detected among 110/1070 confirmatory samples leading to a discordance of 10.3%. It is important to note that out of the final 960 HIV positive infants diagnosed, 828 (86.3%) were diagnosed during first cascade, while 105 (10.9%) and 27 (2.8%) were diagnosed during second and third cascade respectively.

**Fig. 4:**
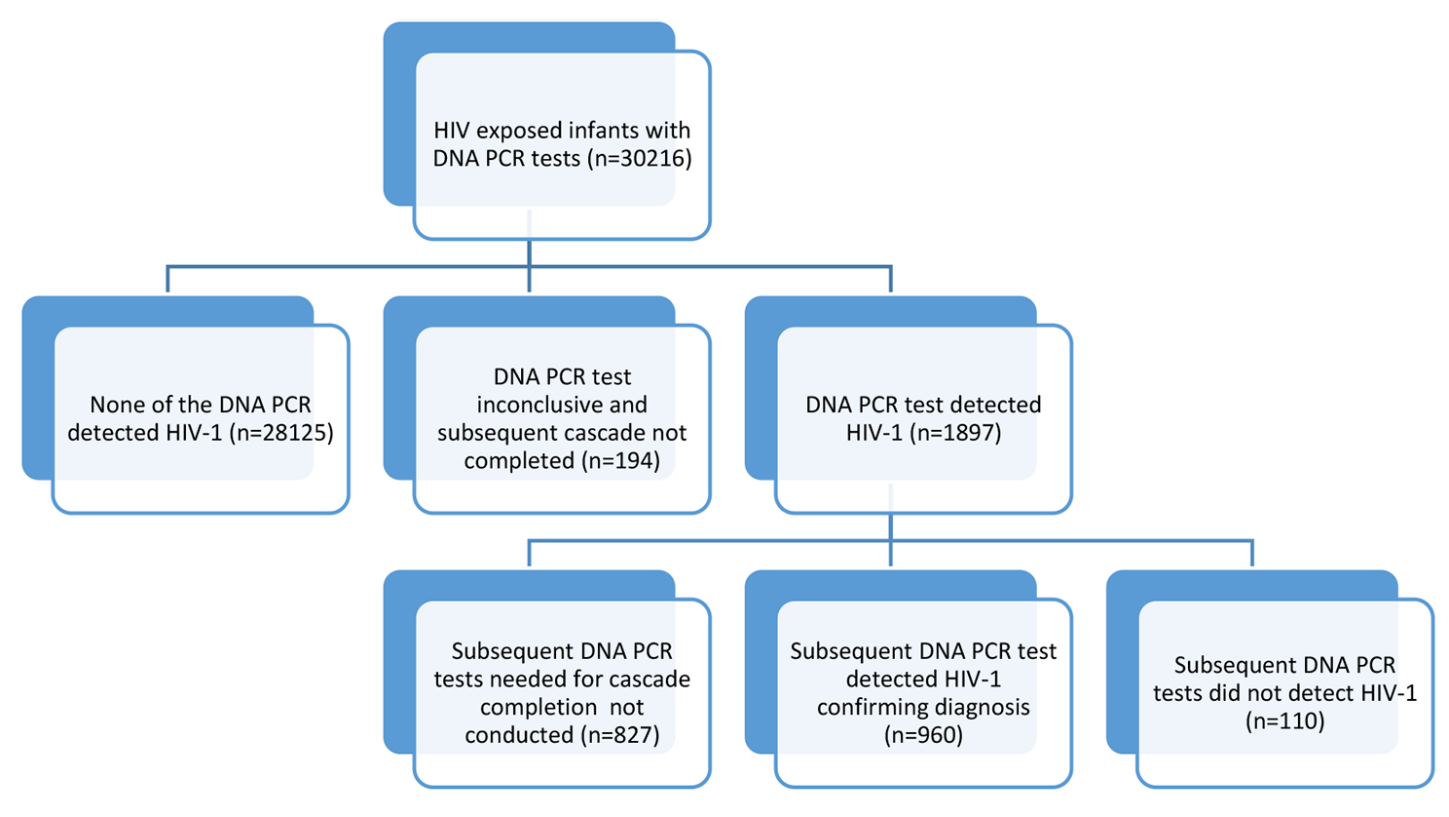
HIV testing outcomes among HIV exposed infants, India (2013–2016)

The overall results of the 30,216 infants can be summarized into five categories (Fig. 4); infants diagnosed with HIV infection (960; 3.2%), those with one sample detecting the virus but not subsequent samples (110; 0.4%), those who did not complete the cascades after detection of HIV in one sample (827; 2.7%), those with inconclusive results (194; 0.6%). The rest 28,125 (93.1%) out of 30,216 infants had no detection of HIV in any of their samples. The ‘age at diagnosis’ data was available for 652 infants born in 2013–2016 period. The overall median age at confirmed HIV diagnosis was 285 days (IQR 174–418 days) and 173 (26.5%) babies had the diagnosis within the first six months. The median age of diagnosis was improved from 352 days (231–467) in 2013 to 181 days (128–256) in 2016. Proportions of children diagnosed within six months of age was also improved from 14.3% for 2013 birth cohort to 49.4% for 2016 birth cohort.

## Discussion

This is first Indian report highlighting the national level data on how early HIV infection is being diagnosed among HIV-exposed infants (HEI) under EID program. It also reflects the status of HEI who were able to complete the cascade in order to confirm their HIV status. The data of all HIV-exposed infants born in India from 2013 to 2016 and had at least one DNA PCR test been presented in the paper. Study has highlighted that overall, 43.6% HEI who detected positive on first sample did not have confirmatory tests (cascade completion). Though, there is ample of evidence [2, 3, 9] on loss to follow-up of EID testing, there is scarcity information on lost to follow-up for confirmatory test in Indian set up as well as rest of the world. Though the reasons for both scenarios may be same, however a more potent approach will be needed to follow these HEI who tested positive on first sample. Such an attrition is of concern; most of these infants were HIV infected and missed opportunity of timely diagnosis and treatment. Community outreach and tracking the HIV-exposed infants is warranted [3].

The findings show that less than half of the infants who had undergone a DNA PCR test had samples collected by 8 weeks of age. The NACP annual report 2015-16 (http://naco.gov.in/sites/default/files/Annual%20Report%202015-16.pdf) reflect that, only 70% infants who had a HIV test under the Early Infant diagnosis program had a DNA PCR test while rest (30%) presented after 6 months of age and were subjected to antibody test. This highlight the delay in collection of first sample. Although, the reporting time for first EID test reduced over the four-year period, it still remains high. Late reporting for giving the first sample delays the whole cascade and strategies are needed to follow-up the mother-baby pair and decentralise sample collection. Optimal decentralisation of sample collection is crucial to achieve this [8]. Under the NACP, HIV viral load testing laboratories can be upgraded for decentralised EID testing in order to collect samples on time and to reduce the turn-around-time (TAT).

The virus detection rate in the first sample was highest in group aged 6 months or more which can be explained because of screening at SA-ICTC with antibody tests. For infants more than 6 months, DBS sample for DNA PCR is collected only if the antibody test is reactive. However, within the first six months, the virus detection rate increased over period of time. We see three possible explanations for this. First, it may be due to delay in reporting for EID testing either due to stigma and discrimination around HIV status of infant [9] or delay in reporting to EID testing ICTC for those children who are ill. It is possible that those who did not get adequate antiretroviral prophylaxis presented late to the health systems even for infant diagnosis. Second possibility is related to the ability of the test to detect the virus during exposure to maternal antiretroviral drugs or Antiretroviral (ARV) prophylaxis. Recent literature notes that ARV exposure during antenatal and postnatal period may result in HIV virological suppression making difficulty in detecting viral material during PCR assays in earlier age of life [10]. Third the most possible explanation is due to post-natal HIV transmission out of breast feeding practices in Indian set up. There may be selection bias with regard to older children who reported to the facility for testing only after developing signs and symptoms of HIV infection.

Fifteen percent  (15%) of the infants with confirmed HIV-1 detection were diagnosed in second or third cascade; their initial DNA PCRs were negative. This highlights the third possibility of ARV drug exposure discussed above. It seems that for these infants the viral loads were too low for detection during the first DNA PCR test or they might have acquired HIV infection through breastfeeding later on. The viral load would have increased to detectable levels during second/third cascade. Even after stopping ARV to the infant, some amount of anti-retroviral drugs would pass through breast milk to the baby. Hence, the strategy to test at 6 months, 12 months and 6 weeks after stopping breast feeding has relevance due to the possibility of false negative results and needs to be continued [11].

This study found that nearly 10% of the infants with HIV-1 detected in first sample, no virus was detected in the second and third DBS samples. False positive results are also likely with lower prevalence. In India, the EID testing is performed using the Amplicor ® HIV-1 DNA Test, version 1.5 by Roche, USA which involved the manual sample processing followed by PCR. The samples are handled separately to reduce any cross contamination. However, the sample integrity and less amount of blood collected on the DBS cards are some of the factors that can contribute to negative results whereas mixing of samples with positive samples as well as mislabeling of samples at the collection site could be possible factors for false positivity of the first sample. However, the discordant samples are being further confirmed during the next follow-up visits. This phenomenon has been reported also in newer interventions with sample collection in first week [12]. Indeterminate range is also a concern where current evidence favours repeat testing [13]. This calls for aiming reduction in turn-around-times for test rather than decrease in number of tests.

Option B plus was initiated in India in 2013 but only in three states and it expanded to the rest of the states over time. This could explain relatively high rate of HIV-1 detection in first DBS sample of 6.3%. India adopted ‘test and treat’ approach in 2017 and expanded access to HIV testing for the pregnant women which is likely to reduce the transmission rates.

The median age of HIV diagnosis was 285 days among HEI in India and only 26.5% children were able to diagnose within first six month of age. Although by 2016, the age at sample collection and subsequent turn-around-time for confirmatory test improved, the median age at first sample was within 2 months whereas the median time at diagnosis remained around 6 months. This time lag between sample collection and sample testing is the crux for delayed HIV diagnosis. Decentralisation of DNA PCR testing at state levels is crucial to reduce the turn-around time and decentralisation of the DBS sample collection shall help in samples collected earlier. Point-of-care testing is being under consideration but the low prevalence setting in India shall pose an eminent challenge. The prevalence of HIV among pregnant women is very low, hence the number of HIV-exposed infants at peripheral facilities are very few. Due to better ART, the mother-to-child transmission is likely to decline significantly and that may pose further challenges to POC strategy. Even a highly specific test may struggle given poor positive predictive value due to very low prevalence. However, POC offers value in cutting down the turn-around-times and need to be tested in an Indian context, given their potential to diagnose early. The HIV epidemic in India is concentrated at few districts. This data from 2013 to 2016 shows that 40% of the cases were from 35 districts out of 700 plus districts. These districts could be focused for better implementation of existing strategies as well as implementation research for POC and similar strategies. The model of testing may be different in districts with high case load and those with low caseload [14].

The study had certain limitations. Since the dataset at RRL level did not include antibody tests done among infants, infants who reported back at 6, 12 and 18 months cannot be confirmed from this study finding. Hence, we are unable to comment on overall retention within EID cascades. Birthdate details were not available for many infants due to poor data quality within the program settings which need to improve. Currently, infant tracking data is being maintained in the program including both antibody and DNA PCR results for HIV-exposed infants which was not the case in 2013-16. Nevertheless, this study is the first one which calculated median age at sample collection and at HIV diagnosis in India for HIV-exposed infants from birth cohorts 2013 to 2016. It also highlights significant attrition from the testing cascades resulting in missed opportunity for diagnosis and treatment of HIV infected infants.

## Electronic supplementary material

Below is the link to the electronic supplementary material.

Supplementary Material 1

Supplementary Material 2

Supplementary Material 3

## Data Availability

The datasets generated and/or analysed during the current study are not publicly available due data accessibility and confidentiality policies of National AIDS Control program, India but de-identified datasets are available from the corresponding author on reasonable request.

## List of abbreviations

ARV: Antiretroviral.
ART: Antiretroviral Therapy.
DBS: Dried Blood Spot samples.
DNA PCR: Deoxyribonucleic Acid Polymerase Chain Reaction.
EID: Early Infant Diagnosis.
HEI: HIV-exposed Infant.
HIV: Human Immunodeficiency Virus.
NACP: National AIDS Control Programme.
RRL: Regional Reference Laboratories.
SA-ICTCs: Stand-Alone Integrated Counselling and Testing Centres.
TAT: Turn-around-time.
WHO: World Health Organisation.
