# Supplementary material for: Loss to follow-up of HIV-exposed infants for confirmatory HIV test under Early Infant Diagnosis program in India: analysis of national-level data from reference laboratories
Source: BMC Pediatr. 2022 Oct 18;22:602. doi: 10.1186/s12887-022-03656-w (PMC9578277; doi:10.1186/s12887-022-03656-w)
Supplement: Supplementary file 2 — Supplementary Material 2 [file 12887_2022_3656_MOESM2_ESM.docx]

**Additional File 2: Diagnostic cascade for second DNA PCR test (n = 10233)**
