# Supplementary material for: Loss to follow-up of HIV-exposed infants for confirmatory HIV test under Early Infant Diagnosis program in India: analysis of national-level data from reference laboratories
Source: BMC Pediatr. 2022 Oct 18;22:602. doi: 10.1186/s12887-022-03656-w (PMC9578277; doi:10.1186/s12887-022-03656-w)
Supplement: Supplementary file 3 — Supplementary Material 3 [file 12887_2022_3656_MOESM3_ESM.docx]

**Additional File 3: Diagnostic cascade for third DNA PCR test (n = 2330)**
